# Outcomes of a tertiary-based innovative approach to engage primary care providers in provision of hepatitis C treatment in community settings

**DOI:** 10.1186/s12889-019-7604-5

**Published:** 2019-10-22

**Authors:** Davoud Pourmarzi, Hayley Thompson, James A. Thomas, Lisa Hall, Andrew Smirnov, Gerard FitzGerald, Tony Rahman

**Affiliations:** 1 School of Public Health and Social Work, Faculty of Health, Institute of Health and Biomedical Innovation (IHBI), Queensland University of Technology, Victoria Park Road, Kelvin Grove, Brisbane, Queensland 4059 Australia; 2 Department of Gastroenterology and Hepatology, The Prince Charles Hospital, Brisbane, 4059 Australia; 3 Department of Gastroenterology and Hepatology, The Prince Charles Hospital, Brisbane, Australia; 4 Faculty of Medicine, The University of Queensland, Brisbane, Australia; 5 School of Nursing, Faculty of Health, Queensland University of Technology, Brisbane, Australia

**Keywords:** Hepatitis C, DAA, Primary care, Community-based

## Abstract

**Background:**

Australia is committed to eliminating the hepatitis C virus (HCV) by 2030. Despite regulations in Australia that enable the prescription of subsidised direct acting antiviral (DAA) by primary health care providers, the number of providers who treat patients for HCV remains low and this limits the prospect of HCV elimination. The Prince Charles Hospital, Brisbane, Australia, implemented an innovative program called Cure-It aimed at engaging primary care providers in community-based HCV treatment. This paper aims to describe initial experiences and short-term patient outcomes of this program.

**Methods:**

A formative evaluation was conducted using program data for the period March 2016 to April 2018. Descriptive statistics were used to report the number of engaged primary care providers, patients’ baseline characteristics, treatment plans, and treatment outcomes.

**Results:**

Thirty primary care providers from different settings were engaged in HCV treatment. Among 331 patients eligible for community-based treatment, 315 (95.2%) commenced treatment, the completion rate was 92.4 and 66.5% achieved sustained virological response at 12 weeks (SVR12). The SVR12 had not been documented for 26.8% of patients. Among patients whose SVR12 was documented, 98.2% achieved SVR12. Only 1.3% of patients experienced treatment failure.

**Conclusion:**

A flexible tertiary-led model can improve primary care providers and patients’ engagement with provision of HCV treatment. Tertiary centres need to play their role to improve the accessibility of HCV treatment through providing training and on-going support for primary care providers while enabling those providers to become more confident in providing treatment independently.

## Background

In Australia, at the end of 2017, an estimated 200,000 people were living with chronic hepatitis C virus (HCV) infection and almost 10,000 had HCV related cirrhosis [1]. The introduction of direct-acting antiviral drugs (DAAs) with their greater efficacy, shorter treatment duration and fewer side effects compared with interferon-based treatment regimens, provides an opportunity to eliminate HCV as a public health threat [2, 3]. The World Health Organisation (WHO) has set a target to eliminate HCV as a public health threat by 2030. Specific targets set by the WHO include increasing HCV treatment coverage from about 1% of affected individuals in 2015 to 80% in 2030 [2, 3].

To help further this aspirational goal, DAAs were listed in the Australian Pharmaceutical Benefits Scheme (PBS) in March 2016, allowing subsidised access to treatment for all patients with HCV. Specialists, “experienced” general practitioners (GPs) and nurse practitioners (NPs) can prescribe DAAs. “Experienced” is defined as participating in a formal education session and managing at least ten patients in consultation with an experienced hepatology, gastroenterology, or infectious diseases specialist [4]. GPs and NPs who are not experienced in HCV management need to consult with a specialist to prescribe DAAs. To receive specialists’ advice and approval for HCV treatment initiation, primary care providers need to submit a ‘remote consultation form’ to specialists [4].

Although this policy aimed to engage Australian primary care providers in provision of HCV treatment, there are still few GPs and NPs providing HCV treatment, and most continue to refer patients to hospitals [5, 6]. The number of patients prescribed DAAs by GPs did not change between 2017 and 2018, and the number who received HCV treatment is falling below the number required to achieve the goal of HCV elimination in Australia by 2030 [7].

New models of care for HCV treatment are needed, which engage more primary care providers and thus increase accessibility by transferring HCV treatment into community settings [3, 4, 8]. To facilitate this, hospitals may need to play a role in training and providing ongoing support for primary care providers.

The Prince Charles Hospital (TPCH), Brisbane, Australia, is a tertiary referral hospital which in 2016 implemented an innovative model called Cure-It aimed at engaging primary care providers in community-based HCV treatment. This paper aims to describe the model of care and to evaluate its initial experience and short term patient outcomes.

### Model of care description

The Cure-It program sought to engage community health centres and general practices in the Metro North area of Brisbane in the provision of HCV treatment. The prevalence of chronic HCV in this region is estimated to be 0.89% of whom 38.3% are diagnosed and 16.2% have received treatment [9].

The program provides training and ongoing support for primary care providers, to empower them to undertake HCV management in community settings. The program was implemented in March 2016 following the availability of DAA therapies on the PBS and associated changes to prescribing requirements which allowed GPs and NPs to prescribe DAAs in consultation with a specialist experienced in HCV treatment. The service is a partnership between TPCH, Brisbane North Primary Health Network and a government-funded drug and alcohol organisation. A hepatology Clinical Nurse Consultant (CNC) facilitated communication between hospital and primary care providers throughout the progam.

The Cure-It program team applied different methods to make GPs in community settings aware of the program. In this regard, information about the program was sent by email and post to practising GPs in the Brisbane Metro North area. The program was advertised in GP targeted newsletters, and the team conducted some educational meetings with GPs at their practices (at 9 general practices) and at other venues. The Education content delivered to GPs was in the form of presentations at 14 meetings delivered by a Clinician experienced in the treatment of HCV. The average of the duration of the meetings was 1 h and on average, 6 GPs attended each meeting. Education covered basic pathophysiology, assessment of HCV infectivity, risk factors for acquisition, fibrosis assessment, treatment options and assessment of successful treatment. Education was further reinforced with the provision of written information, posters, newsletters and email notifications.

Current Australian national guidelines, note that ‘DAA treatment naïve’ patients without decompensated liver disease, renal failure, HIV coinfection, or hepatitis B coinfection are eligible to be treated in community settings [4, 10]. The program assessed the patient’s eligibility for community-based treatment based on the patient’s situation and the primary care provider’s ability and experience in HCV treatment. Potential drug interactions were checked using the University of Liverpool HEP drug interaction website. Disease extent was assessed based on Aspartate Aminotransferase to Platelet Ratio Index (APRI) or FibroScan® results. Patients with APRI < 1 or liver stiffness < 12.5 kPa were considered as patients with low probabibilty of advanced fibrosis or cirrhosis. Other factors such as platelet count, synthetic liver function and +/− ultrasound results and duration of infection were also factored into decision making by the Hepatologist.

The GPs were engaged in the program, either through review of the treatment waiting list by the program team or by direct approach from the GP. The list of patients waiting for treatment at TPCH was reviewed and eligible patients for community-based treatment were identified. A specialist reviewed the documents and provided advice on eligibility. The GP who referred the patient was contacted to discuss the possibility of HCV treatment in their practice with training and support from the hospital. If GPs agreed to initiate the treatment, the CNC would provide initial information and send the pre-filled remote consultation form and asked GPs to fill it out and order tests as required and send it back to the CNC.

Additionally, primary care providers who were aware of the program sent the remote consultation form to the hospital or contacted the hospital directly via email or phone to receive approval.

In all cases, the Hepatologist assessed the patient’s eligibility for community-based treatment based on clinical information provided in the remote consultation form. If the patient was eligible, the specialist provided advice on the medication regimen, treatment duration and side effects monitoring along with further investigations required. The CNC then discussed the treatment plan with the primary care providers and provided the specialist’s advice along with a direct contact number for advice.

Current Australian recommendations do not require treatment monitoring by way of pathology testing [4]. Case managers within the drug and alcohol settings and practice nurses or other staff within general practices contacted some patients who required more active monitoring. These patients were identified based on the individual primary care provider’s assessments and the program team were not involved in this process. Data on this was not collected.

To monitor the effectiveness of the program, primary care providers reported patients who had achieved sustained virological response (SVR), failed treatment, or re-acquired HCV. Additionally, the Hepatology CNC monitored treatment response periodically through follow up of patients’ pathology results.

## Methods

This study is a formative evaluation which collects and analyses administrative data. Thus it is an evaluation based on collected data of a hospital based program and not a research project in its own right [11].

Remote consultation, specialist response and the patient’s follow up data were collected during the program by the TPCH Department of Gastroenterology and Hepatology. Data of general practice postcode, patient age, sex, data of diagnosis, cirrhosis status, antipsychotics use, current or recent drug use, alcohol use > 40 g/day, receiving OST, HIV and HBV co-infection, prior HCV treatment, FibroScane results, and APRI were provided by primary care providers using remote consultation form. The prescribed treatment and treatment duration was collected from specialist response to primary care providers request in remote consultation form. Data on treatment outcome (SVR, failed treatment, and re-acquired HCV) were either provided by primary care providers to program team or through follow up of patients’ pathology results by CNC.

For this study, data from March 2016 to April 2018 was reviewed retrospectively. Patients prescribed DAA by primary care providers were considered as having ‘commenced treatment’ and those who completed the recommended duration of treatment were recorded as ‘completed treatment’. SVR12 achieved patients were those in whom HCV-RNA was undetectable 12 weeks or more after completing treatment. Patients who did not attend HCV PCR test 12 weeks or more after completing treatment were considered as non-attenders for SVR12.

Descriptive statistics were used to report the number of primary care providers engaged, patients’ characteristics, treatment plan, and treatment outcomes. Data were analysed with consideration of all eligible patients for community-based treatment who were alive to start the treatment. Data were analysed using STATA 15. To describe the program’s geographical coverage, the Queensland Hospital and Health Service Referral map in ArcGIS® 10.6 was used based on the involved primary care providers’ practice suburb name.

## Results

Between March 2016 and April 2018, one Addiction Specialist, one NP and four GPs from four different drug and alcohol services and 24 GPs practising in private practices used the training and support provided through the Cure-It program to initiate HCV treatment. Eleven primary care providers directly approached the program team to receive training and support to initiate treatment in community settings. In total, 21 GPs were approached after they referred patients to the hospital and two of them (9.5%) refused to provide HCV treatment.

**Fig. 1:**
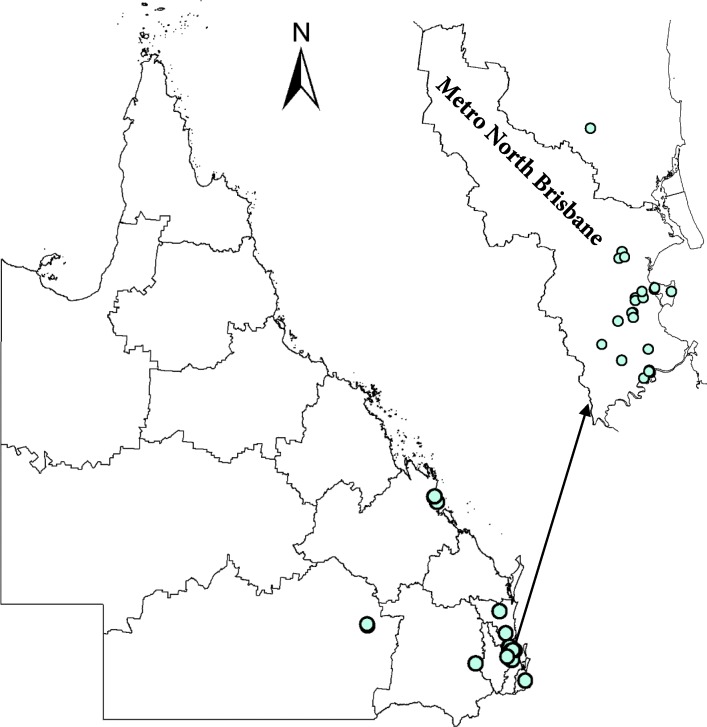
Geographically distribution of engaged primary care providers in Cure-It program

Through supporting primary care providers in drug and alcohol services the program provided opportunities to engage with ‘hard to reach’ populations such as people who inject drugs. Three of these services were collocated with needle and syringe programs, one was also collocated with sexual health services. Although most of the engaged GPs were practising in Metro North Brisbane, the program increased accessibility of HCV treatment across different regions of Queensland. (Fig. 1).

Among 343 remote consultations, 333 patients were eligible to be treated in community settings (97.1%), of whom two people died for reasons unrelated to HCV before commencing treatment. Among 331 patients eligible for community-based treatment, 315 (95.2%) commenced treatment. The mean age of patients was 44.6 ± 10.4 years (range: 23–95).

In 166 patients the date of diagnosis was known and the mean time from diagnoses to treatment was 13.9 ± 9.6 years (range: 1–48). The majority of patients were male (67.3%) and received treatment from drug and alcohol services (87.9%). Alcohol use > 40 g/day was reported in 28 (8.9%) of the patients and two (0.6%) were HCV and HBV co-infected. About 36% were prescribed Sofosbuvir plus Daclatasvir. The treatment duration for most of the patients was 12 weeks (95.2%). (Table 1).

**Table 1:** Characteristics of HCV treatment commenced patients in community settings

Variables		N (%)
Sex (*n* = 315)	Male	212 (67.3)
	Female	103 (32.7)
HCV genotype (n = 315)	1	156 (49.7)^a^
	2	9 (2.9)
	3	148 (47.1)
	4	1 (0.3)
	6	2 (0.6)
Age	≤30	27 (8.6)
	31–40	87 (27.6)
	41–50	112 (35.6)
	51–60	70 (22.2)
	> 60	19 (6.0)
Currently or recently drugs use^b^ (*n* = 225)	Yes	95 (44.2)
	No	130 (57.8)
Receiving OST (*n* = 314)	Yes	87 (27.7)
	No	227 (72.3)
Antipsychotics use (n = 315)	Yes	111 (35.2)
	No	204 (64.8)
Prior HCV treatment	Peg-IFN or IFN plus Rib	7 (2.2)
	DAA	6 (1.9)
	No	302 (95.9)
Cirrhotic patients	Yes	29 (9.2)
	No	286 (90.8)
FibroScan ≥ 12.5 kPa (*n* = 112)	Yes	4 (3.6)
	No	108 (96.4)
APRI ≥ 1 (*n* = 308)	Yes	46 (14.9)
	No	262 (85.1)
Prescribed DAA (n = 314)	Sofosbuvir plus Daclatasvir	112 (35.7)
	Sofosbuvir plus Ledipasvir	95 (30.2)
	Epclusa	62 (19.7)
	Zepatier	31 (9.9)
	Sofosbuvir plus Velpatasvir	10 (3.2)
	Paritaprevir/ritonavir plus Ombitasvir plus Dasabuvir	3 (1)
	Elbasvir/Grazoprevir	1 (0.3)
Treatment duration (*n* = 315)	8 weeks	3 (1)
	12 weeks	300 (95.2)
	24 weeks	12 (3.8)

^a^one patient with genotype 1 and 3, one patient with genotype 1 and 6, and one patient with undetected genotype

^b^: Currently using drug or used drug within 6 months before the treatment

*OST* opioid substitute treatment, *APRI* Aspartate Aminotransferase to Platelet Ratio Index

Peg-IFN: Pegylated interferon

*IFN* interferon

*Rib* ribavirin

**Fig. 2:**
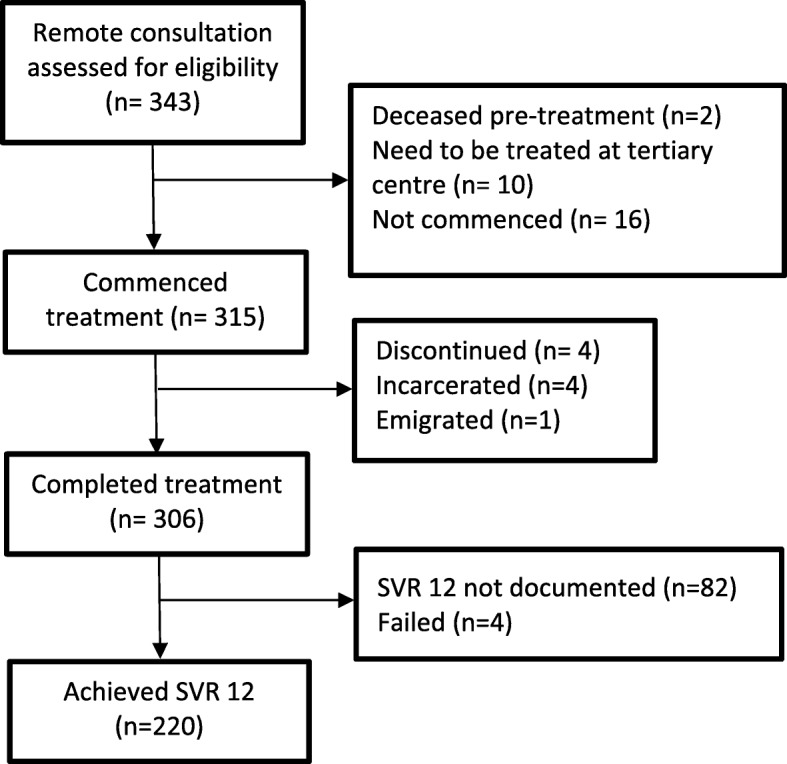
Patient disposition

Of 315 patients who commenced treatment, 306 (92.4%) completed the treatment plan and 220 (66.5%) achieved SVR 12. SVR12 had not been documented for 26.8% of patients, however, seven tested negative at the end of treatment (end of treatment response). Among patients whose SVR12 was documented, 98.2% achieved SVR12. The SVR12 was achieved in 95.6% of patients who receive treatment at general practices and 98.5% of patients who receive treatment at drug and alcohol services. Treatment failure was reported in only 1.3% of patients. (Fig. 2).

## Discussion

The Cure-It program improved the accessibility of HCV treatment by engaging primary care providers practicing in different community settings. More than 90% of GPs who were approached agreed to provide HCV treatment. The high uptake (95.2%) and treatment completion (92.4%) rates and low treatment failure rate (1.3%) demonstrate the potential for providing acceptable and clinically effective treatment in community settings with the support of a tertiary-led program.

Although primary care providers can prescribe DAAs, there are many barriers that limit their involvement in HCV treatment. Lack of knowledge among primary care providers about HCV and patients’ eligibility for DAA treatment, and an undeveloped pathway to gain experience in HCV treatment, are some of the barriers highlighted in the Australian context [5, 6, 12, 13]. The program implemented at TPCH attempted to address these barriers and change the traditional direction of the referral pathway.

In this program, a tertiary centre offered training and ongoing support to primary care providers to provide HCV treatment locally. The program endeavoured to build friendly ongoing relationships with the primary care providers and be easily accessible and approachable. This prevented unnecessary hospital attendance and helped to overcome many barriers related to tertiary-based treatment accessibility [12].

For primary care providers to be involved in the provision of HCV treatment, they need to be confident that they can access sufficient professional support when needed [14]. Making primary care providers aware of ‘easy to access’ support was one of the operational elements of this program. In a survey on 191 GPs in Victoria, Australia, 21% of GPs reported that they tried to receive specialist approval to prescribe DAA, but only 13% of GPs believed that support was available and 12.5% were satisfied with the process [5]. Other studies also found an increase in primary care providers’ involvement with HCV treatment as a result of providing training and support from tertiary centres [15–18].

The high uptake and treatment completion rates and low confirmed treatment failure rate, implies the acceptability and clinical effectiveness of a tertiary-led model for provision of HCV treatment in community settings providing HCV treatment in community settings. Similar uptake and completion rates have been reported from other community-based models of care for HCV treatment. A study of a remote consultation pathway to support GPs to prescribe HCV treatment in Victoria, Australia, showed that 83% of approved patients for GP treatment commenced treatment [19]. In a randomised clinical trial in USA, of patients who agreed to participate, 93% initiated the treatment and 82% completed treatment [20]. In two other studies, 97% [21] and 96% [22] of patients attending a drug and alcohol service completed the treatment. The treatment failure rate of 1.3% in our study was lower than the 4.5% [21] and 6.6% [20] failure rates reported on DAA treatment for HCV in community settings.

We found a considerable number of patients (26.8%) whose SVR12 were not documented at the time of analysis. As considerable efforts were made by the hospital team to identify SVR12 results, it is likely that these patients did not attend an SVR12 appointment. This may be attributable to the personal circumstances of the patients or to a relative lack of follow-up and encouragement to be retested. This problem was also reported in another study involving remote consultation in which SVR12 for more than half of eligible patients was not documented [19]. In studies where the data were specifically collected for the purpose of the research, the percentage of non-attending patients for SVR12 was lower and ranged from 1.5 to 8.2% [20–22]. The difference between data collected in routine practice and research contexts showed the importance of providing intensive follow-up for some patients.

About 30% of patients who commenced treatment through this program were currently or recently engaged in drug use and 28% were receiving opioid substitution therapy. Supporting primary care providers in drug and alcohol services and collocation with needle and syringe programs and sexual health services improved the accessibility of HCV treatment for ‘hard to reach’ populations, and provided opportunities to engage with the target population, which are critical for HCV elimination in Australia [23, 24]. As there are many barriers that prevent this group of people accessing tertiary-based services, to increase treatment access it is essential to provide this treatment through primary care providers in varied settings [12, 24].

### Strengths and limitations

This study is one of the first to report real-world outcomes of a tertiary-led innovation to support community-based HCV treatment in the DAA era. The retrospective manner of data collection is a limitation. Although various strategies were used to follow up patients’ for SVR12, there was a considerable number of patients for whom SVR12 status was not confirmed.

## Conclusion

The results of this study demonstrate that a flexible tertiary-led model can improve the accessibility of HCV treatment in community settings through engaging primary care providers. The high rate of treatment uptake and completion and the low rate of treatment failure supports the acceptability and clinical effectiveness of HCV treatment by primary care providers. Involving tertiary centres to provide training and on-going support for primary care providers through programs like Cure-It may lead to the sustainable provision of treatment in community settings. Further studies are required to identify and better understand the barriers and enablers to providing HCV treatment in community settings, and also the experience of primary care providers and patients with community-based HCV treatment.

## Data Availability

Data is available from the corresponding author under reasonable request with permission from the Prince Charles Hospital.

## Abbreviations

APRI: Aspartate Aminotransferase to Platelet Ratio Index
CNC: Clinical Nurse Consultant
DAAs: Direct-acting antivirals
GP: General practitioner
HCV: Hepatitis C virus
IFN: interferon
NP: Nurse practitioner
OST: opioid substitute treatment
PBS: Pharmaceutical Benefits Scheme
Peg-IFN: Pegylated interferon
Rib: ribavirin
SVR: Sustained virological response
TPCH: The Prince Charles Hospital
WHO: World Health Organisation
